# Intrauterine growth restriction in piglets alters blood cell counts and impairs cytokine responses in peripheral mononuclear cells 24 days post-partum

**DOI:** 10.1038/s41598-020-61623-w

**Published:** 2020-03-13

**Authors:** Charlotte Amdi, Julie C. Lynegaard, Thomas Thymann, Andrew R. Williams

**Affiliations:** Department of Veterinary and Animal Sciences, Faculty of Health and Medical Sciences, University of Copenhagen, DK-1870 Frederiksberg C, Denmark

**Keywords:** Haematopoiesis, Intrauterine growth

## Abstract

Large litter sizes have resulted in more piglets being exposed to intrauterine growth restriction (IUGR). Growth restriction during fetal life is linked with lower growth efficiency and increased susceptibility to infections in postnatal life and IUGR may associate with an altered innate immune system. We investigated the haematological, thromboelastography and plasma biochemical profiles of IUGR and normal piglets as well as cytokine responses in peripheral blood mononuclear cells stimulated with lipopolysaccharide (LPS) at 24 days of age. Piglets were classified at birth based on their head morphology as either IUGR or normal. The present study showed a modulation of the immune function of IUGR pigs, characterized by an increase in neutrophil percentage and fibrinogen levels but a decrease in CD4+ T-cells. A lower level of LPS-induced IL-1β production was evident in IUGR pigs, suggesting immunological hypo-responsiveness. Furthermore, higher levels of reticulocytes, MCV and MCH and lower levels of erythrocytes in IUGR pigs suggest altered bone marrow hematopoiesis. All together, the results suggest a moderate suppression of the immune response of IUGR piglets, which may have implications for resistance to pathogen challenges in the post-weaning period. Serum metabolites and blood clotting profile did not differ between IUGR and normal piglets.

## Introduction

Danish sows produce the largest piglet litters in the world. Whilst this has benefits for pig producers in terms of production efficiency, there is also a well-known correlation between the number of piglets in a litter and the number of pigs that are small for gestational age or intrauterine growth restricted (IUGR)^1^. One major consequence of growth restriction is an increased perinatal piglet mortality. Moreover, piglets that are exposed to different degrees of IUGR, have altered fetal growth patterns, with nutrients being preferentially partitioned to the brain rather than other tissues (brain sparing effect)^2^. This further makes IUGR piglets more challenged at birth^3^ and it is estimated that up to 30% of piglets show signs of IUGR in Danish litters^4,5^. In addition, large litters result in piglets that are smaller and have less mature immune and digestive systems at weaning^6,7^.

Pigs exposed to IUGR also show lower postnatal growth velocity compared to normal weight littermates^8^, however, it is not known how IUGR affects the postnatal immune response relative to normal –weight pigs. Because of the diversion of nutrients away from growth in support of immune-related processes, immune challenge is considered a major obstacle to animals’ achieving their genetic potential for growth or efficiency of gain^9^. IUGR piglets may be more susceptible to disease, as their growth is already compromised, and thus more nutrients are portioned to immune defense.

The aim of this study was to characterize the peripheral immune phenotype and function in IUGR piglets, specifically by quantifying whole blood and lymphocyte subsets and the cytokine response in PBMC following stimulation with the proinflammatory agent lipopolysaccharide (LPS), i.e. a major component of the cell wall of gram-negative bacteria. To further document the influence of IUGR, we measured thromboelastography (TEG) as this represents a cascade of reactions during blood coagulation which is intimately linked to the innate immune system. Finally, a plasma profile of relevant biochemical markers were measured. It was hypothesized that TEG results in pigs would identify abnormalities/differences and a disruption of hemostasis between the two groups of pigs. Our overall hypothesis was that IUGR piglets at the age of 24 days would have an altered immune phenotype compared to normal piglets.

## Methods

### Animals and experimental design

Forty piglets (20 IUGR and 20 normal) were selected from a 1,600-sow (Danish Landrace × Danish Yorkshire) Danish piggery. The piglets were a subpopulation of pigs in a trial investigating growth, whole blood glucose and rectal temperatures of IUGR piglets compared to normal piglets^10^. Piglets were selected on the day of birth (day 0), before litter equalization, and ear tagged, and sex of the piglet, sow identification, litter size, and birth weights (BiW) were noted. Piglets were visually graded and categorized as either IUGR or normal by the modified characteristics from Hales *et al*.^5^ and Chevaux *et al*.^11^. Piglets defined as IUGR pigs included the criteria: (a) a steep dolphin-like forehead, and one or more of the following characteristics: (b) bulging eyes, (c) wrinkles perpendicular to the mouth or (d) hair with no direction of growth. If none of the criteria applied, the piglet was defined as normal. For each IUGR piglet in a litter, one corresponding normal piglet (none of the criteria) was chosen from the same litter. The piglets were individually weighed on a scale (UWE, Bjerringbro Vægte ApS, Bjerringbro, Denmark) and ear tagged for identification.

### Housing, feeding and management

All experimental animals were managed according to general routines at the farm. While in the farrowing unit, piglets were housed with a sow in individual farrowing pens (1.67 m × 2.38 m), with slatted floor (1.3 m × 1.67 m) and a floor heated creep area (0.60 m^2^). Litters for both sows and gilts were equalized to 16 piglets by cross-fostering piglets born within the same 12 to 24 hours. No castrations were performed in the production and the experiment therefore included entire males. The sows had ad libitum access to drinking water and were fed a commercially formulated mash diet according to Danish recommendations based on wheat, barley and soybean meal (Vilomix A/S, Mørke, Denmark). In addition, all farrowing pens were installed with automatic milk cups, where the piglets had access to milk replacer (Schils, Sittard, the Netherlands) from day one. Three to four times daily from day 14, the piglets received a handful dry feed from a commercial mix (Danish New Wean feed, Danish Agro, Karise, Denmark).

### Blood and tissue sampling

On day 24, piglets were transported to research facilities at the University of Copenhagen and blood sampled within an hour after arriving to the facilities. In order to process the samples as quickly as possible (within an hour), piglets were brought in over four consecutive weeks (5 IUGR and 5 normal pigs per week). Five blood samples were taken from each piglet. The first sample was taken in a 1.8 ml citrat stabilized tube for (TEG) and fibrinogen analysis. The second and third samples were taken into heparinized tubes for the LPS challenge on blood, and phenotyping of peripheral leukocytes by flow cytometry. The fourth sample was taken in a 4.0 ml tube (EDTA) for hematology (CBC/Diff/Retic) and finally the fifth sample was taken in a 4.0 ml tube for serum, spun down and serum frozen for later analysis of IGF-1 and biochemistry. The tubes for TEG were taken to a nearby lab for immediate processing and likewise the tubes for the LPS challenge.

### Blood sample analysis

The TEG analyses were performed with the same model of a computerized thromboelastograph (TEG 5000 Haemostasis Analyzer, Haemoscope Corporation, Niles, IL) according to the previously published method^12,13^. In brief, data were obtained continuously by electronic transfer to computer from the analyzer. The hematology profile was analyzed on an Advia 2120 Hematology System (Siemens Healthcare Diagnostics, Tarrytown, NY, USA) and the serum analyses for biochemistry were assayed using an Advia 1800 Chemistry System (Siemens Healthcare Diagnostics, Tarrytown, NY, USA).

### LPS challenge

Peripheral blood mononuclear cells (PBMC) were obtained from heparinized blood samples by differential centrifugation using histopaque 1.077 (Sigma-Aldrich). Cells were washed, counted and seeded at 5 × 10^6^ cells/mL in RPMI 1640 media supplemented with 2 mM L-glutamine, 10% heat-inactivated foetal calf serum, 100 U/mL penicillin and 100 µg/mL streptomycin. PBMC were then stimulated with either 1 µg/mL LPS (*Escherichia coli* O55:B5; Sigma-Aldrich) or mock treated with PBS as controls. The PBMC were then cultured for 24 hours at 37 °C and 5% CO_2_, before the supernatant was harvested and stored at −20 °C. Cytokine concentrations were then measured by ELISA using commercial antibody pairs according to the manufacturer’s instructions (IL-10 and TNFα – ThermoFisher; IL-6, IL-8, IL-1β – Duosets, R and D Systems).

### Flow cytometry

PBMC were isolated as above, washed and surface stained with either mouse anti-porcine CD3 (clone BB23-8E6-8C8; BD Biosciences), CD4 (clone 74-12-4; BD Biosciences), CD8 (clone 76-2-11; BD Biosciences) or γδ TCR (clone PGBL22A; VWR). Alternatively, cells were prepared for intracellular staining using a fixation/permeabilisation kit (BD biosciences) and stained with mouse anti-human CD79α. Appropriate isotype controls were included. Cells were then analysed with an Accuri C6 flow cytometer (BD biosciences). Lymphocytes were gated based on forward/side scatter and 10,000 events collected within the gate. Gating strategies used to define CD3^+^CD4^+^ and CD3^+^ CD8^+^ single and double-positive cells are shown in Supplementary Fig. 1.

### Statistical analysis

Data was analyzed in the statistical program SAS (GLM procedure of SAS; SAS Inst.Inc., Cary, NC) according to the following model:

$${Y}_{ij}=\mu +{\alpha }_{i}+{\beta }_{j}+{(\alpha \beta )}_{ij}+{\varepsilon }_{ij}$$

where *Y*_*ij*_ is the dependent variable measured (cells, blood characteristics, cytokines, blood parameters), *μ* denotes the overall mean, *α*_*i*_ denotes the effect of classification (i = IUGR, normal), *β*_*j*_ denotes the effect of batch (j = 1, 2, 3, 4), *(αβ)*_*ij*_ is the interaction between classification × batch, and *ε*_*ij*_ describes the random error term. The interaction between classification × batch was only included when significant. Means were separated using the PDIFF option and presented as Least Square Means ± SEM and considered significant when *P* < 0.05 and a tendency when *P* < 0.10.

### Ethics approval and consent to participate

The experiment was carried out with respect to animal experimentation, and with the approval of the Danish Animal Experimentation Inspectorate (2016-15-0201-01018).

## Results

### Blood analysis

The birth weight and body weight at day 24 are presented in Table 1. On average the normal piglets weighed 1.51 ± 0.05 kg at birth and 6.84 ± 0.31 kg at day 24 of age and an IUGR piglet weighed 0.77 ± 0.02 kg and 4.53 ± 0.17 kg at day 24 of age (Table 1; *P* < 0.001). There was a difference between the groups on number of erythrocytes and percentage of lymphocytes (*P* < 0.05) with lower levels found in IUGR piglets compared to normal piglets. Further, there were differences between MCH, MCV, percentage neutrophils and fibrinogen (*P* < 0.05), with lower levels in normal pigs compared to IUGR pigs. IGF-1 levels were similar between groups, with 61.3 ng/ml vs. 67.6 ng/ml (SEM 2.64 ng/ml; *P* = 0.151) in IUGR and normal pigs respectively. The TEG analysis is presented in Table 2. Similar TEG values were obtained between IUGR and normal pigs for most parameters, however IUGR pigs tended to reach lysis at 60 min, but a lower clot lysis at 30 min, compared to normal pigs. No differences were observed in the blood biochemistry of piglets at day 24 of age (Table 3).

**Table 1:** The characteristics and blood profile of IUGR and normal piglets at day 24 of age.

Items	Classification		P-values
	Normal	IUGR	
*n*	20	20	
Birth weight, kg	1.51 ± 0.045	0.77 ± 0.016	0.001*
Body weight at day 24, kg	6.84 ± 0.313	4.53 ± 0.174	**0.001**
*n*	18	18	
Total leukocytes, mia/L	12.31 ± 1.200	10.99 ± 0.871	0.380
Total erythrocytes, bill/L	6.10 ± 0.102	5.44 ± 0.176	**0.003**
Haemoglobin (HGB), mmol/L	7.5 ± 0.11	7.1 ± 0.26	0.231
Hematocrit (HCT), L/L	0.389 ± 0.004	0.372 ± 0.0117	0.163
MCH, fmol	1.23 ± 0.018	1.31 ± 0.017	**0.003**
MCHC, mmol/L	19.19 ± 0.170	19.15 ± 0.172	0.875
Thrombocytes	443 ± 49.3	386 ± 30.0	0.325**
Mean platelet volume (MPV), fL	9.4 ± 0.36	9.7 ± 0.29	0.521
Mean cell volume (MCV), fL	64.0 ± 0.66	68.5 ± 0.77	**0.001**
Mean platelet count, (MPC), g/L	208 ± 3.2	205 ± 2.4	0.372
Neutrophils, pct	55.4 ± 1.81	62.5 ± 2.12	**0.016**
Lymphocytes, pct	39.9 ± 1.68	32.7 ± 1.93	**0.008**
Monocytes, pct	2.3 ± 0.25	2.5 ± 0.41	0.648
Eosinophils, pct	0.9 ± 0.26	1.0 ± 0.27	0.860
Basophils, pct	0.6 ± 0.15	0.4 ± 0.04	0.205
Large unstained cells (LUC), pct	0.8 ± 0.10	0.9 ± 0.13	0.841
Neutrophils, mia/L	6.73 ± 0.554	6.97 ± 0.702	0.784
Lymphocytes, mia/L	4.97 ± 0.644	3.53 ± 0.270	**0.046**
Monocytes, mia/L	0.29 ± 0.039	0.25 ± 0.026	0.427
Eosinophil, mia/L	0.11 ± 0.032	0.10 ± 0.029	0.809
Basophils, mia/L	0.10 ± 0.050	0.05 ± 0.006	0.273
Large unstained cells (LUC), mia/L	0.11 ± 0.026	0.09 ± 0.013	0.437
Reticulocytes, pct (estim)	3.9 ± 0.41	5.0 ± 0.55	0.115
Absolut reticulocyte, mia/L (estim)	236.5 ± 23.09	274.9 ± 29.93	0.317
Fibrinogen, g/L	6.23 ± 0.395	8.05 ± 0.700	**0.035**

*Tendency of batch **Effect of batch.

No interactions between classification and batch were observed.

MCH = mean corpuscular hemoglobin, MCHC = Mean corpuscular hemoglobin concentration,

**Table 2:** Thromboelastography (TEG) of whole blood from IUGR and normal piglets at day 24 of age.

Items	Classification		P-values
	Normal	IUGR	
*n*	17	20	
SP, min	5.6 ± 0.53	5.6 ± 0.40	0.977
R, min	6.6 ± 0.64	6.5 ± 0.45	0.873
K, min	1.6 ± 0.16	1.6 ± 0.10	0.657
Ang°	68.8 ± 1.90	70.0 ± 1.09	0.579
MA, mm	70.3 ± 1.21	72.0 ± 1.45	0.420
Gkd	12.3 ± 0.71	13.8 ± 1.08	0.270
Ly30, %	2.6 ± 0.52	2.7 ± 0.54	0.835
Ly60, %	6.3 ± 0.75	6.7 ± 0.81	0.089*
CL30, %	94.0 ± 0.82	93.6 ± 0.88	0.074*
CL60, %	87.0 ± 1.13	86.5 ± 1.22	0.743**

*Interaction between classification and batch P < 0.05.

**Tendency for an effect of batch P < 0.10.

R, reaction time (time to the clot initiation); K, k value (the time for the tracing to achieve a set clot strength); Ang, angle (the rate of clot formation); MA, maximum amplitude (the greatest clot strength); Ly, lysis; 30, 30 minutes after reaching MA; 60, 60 minutes after reaching MA; CL, clot lysis.

**Table 3:** Effects of serum metabolites and electrolytes at day 24 of age.

Items	Classification		P-values
	Normal	IUGR	
*n*	17	18	
Albumin g/L	34.9 ± 0.79	34.5 ± 1.31	0.796
Total protein, g/L	49.95 ± 1.016	47.65 ± 1.311	0.207
Basic phosphatase, U/L	678.1 ± 38.28	773.8 ± 68.13	0.281
Alanin amino transferase U/L	56.1 ± 2.16	57.9 ± 6.43	0.672
Cholesterol, mmol/L	3.13 ± 0.178	3.02 ± 0.220	0.645
Creatinine, umol/L	80.1 ± 2.75	74.6 ± 2.65	0.176
Creatinine Kinase	576.5 ± 121.58	453.3 ± 37.75	0.347
Iron	21.6 ± 2.51	22.1 ± 2.30	0.998
Inorganic phosphate, mmol/L	3.08 ± 0.053	3.11 ± 0.099	0.815
Aspartate amino transferase U/L	57.6 ± 4.03	64.8 ± 4.22	0.228
Blood urea nitrogen, mmol/L	3.61 ± 0.247	3.64 ± 0.374	0.897
Gamma-glutamyl transferase, U/L	24.4 ± 2.22	24.5 ± 2.69	0.978
Calcium, mmol/L	3.20 ± 0.024	3.18 ± 0.058	0.703
Magnesium, mmol/L	1.05 ± 0.029	1.07 ± 0.034	0.751
Sodium, mmol/L	141.8 ± 0.93	143.2 ± 0.94	0.384
Potassium, mmol/L	4.2 ± 0.12	4.4 ± 0.21	0.427
Glucose, mmol/L	6.3 ± 0.12	6.2 ± 0.38	0.769
Triglyceride, mmol/L	0.95 ± 0.098	0.92 ± 0.096	0.862

No interactions between classification and batch were observed.

### Cytokine and lymphocyte concentrations

The concentrations of cytokines released from LPS-stimulated and mock-treated cells are shown in Table 4. There was a higher concentration of IL-1β following LPS stimulation in PBMC from normal piglets compared to cells from IUGR piglets (17.3 ± 2.55 *vs*. 9.9 ± 1.75 ng/mL; *P* = 0.021). Normal piglets also had numerically higher concentrations of IL-6 (1508 ± 356 *vs*. 930 ± 162 pg/mL; *P* = 0.148) although this difference was not significant. The profile of the lymphocyte population in PBMC from normal and IUGR piglets is shown in Table 5. There was a tendency towards a higher percentage of CD4+ T-cells (49.5 ± 1.82 *vs*. 43.6 ± 2.45; *P* = 0.063) in normal piglets compared to IUGR piglets.

**Table 4:** Cytokine production in peripheral blood mononuclear cells with and without lipopolysaccharide (LPS) stimulation.

Items	Classification		P-values
	Normal	IUGR	
*N*	19	20	
***IL-10 (pg/mL)***			
No LPS	0	2.3 ± 2.34	0.308
LPS	105.6 ± 17.79	104.2 ± 18.15	0.994
***IL-6 (pg/mL)***			
No LPS	0	0	NA
LPS	1508.2 ± 355.80	929.7 ± 162.13	0.148
***IL-8 (ng/mL)***			
No LP	4.4 ± 0.94	5.2 ± 1.55	0.679*
LPS	213.7 ± 26.20	211.4 ± 38.48	0.961
***TNFa (pg/mL)***			
No LPS	123.8 ± 26.22	198.2 ± 81.01	0.419
LPS	1479.0 ± 412.04	1101.2 ± 356.21	0.491
***IL-1β (ng/mL)***			
No LPS	0	0	NA
LPS	17.3 ± 2.55	9.9 ± 1.75	**0.021**

*Effect of batch.

No interactions were observed between batch and classification.

**Table 5:** Percentages of total T-cells (CD3^+^), γδ T-cells, B-cells (CD79α^+^), and CD4^+^ and CD8^+^ cells within the peripheral blood mononuclear cell population, and percentages of CD4^+^ and CD8^+^ T-cells (CD3^+^CD4^+^ and CD3^+^CD8^+^).

Items	Classification		P-values
	Normal	IUGR	
*n*	18	16	
CD3^+^	33.55 ± 1.465	31.34 ± 1.804	0.342
γδ TCR^+^	9.21 ± 0.762	8.65 ± 0.623	0.541
CD79 α ^+^	34.52 ± 2.197	32.27 ± 2.148	0.486
CD4^+^	21.59 ± 1.248	19.10 ± 1.587	0.138*
CD8^+^	31.79 ± 2.386	31.87 ± 3.212	0.934*
CD8^low^	13.52 ± 1.240	14.39 ± 1.575	0.630*
CD8^High^	18.27 ± 1.673	17.48 ± 2.069	0.711
CD4^+^CD8^+^	8.21 ± 0.469	7.79 ± 0.552	0.567
CD3^+^CD4^+^	49.47 ± 1.815	43.56 ± 2.449	**0.063**
CD3^+^CD8^+^	39.78 ± 2.627	40.99 ± 3.080	0.744
CD3^+^CD8^low^	21.81 ± 1.637	22.51 ± 1.136	0.601*
CD3^+^CD8^High^	17.98 ± 1.851	18.48 ± 2.370	0.883
CD3^+^CD4^+^CD8^+^	17.21 ± 1.282	17.24 ± 1.166	0.923*

*Effect of batch

No interactions between classification and batch were observed.

## Discussion

There is a high mortality in IUGR piglets compared to normal piglets during the suckling period in Danish piggeries^5^. In addition, most pigs are challenged at weaning as they are removed from the passive immune protection from the milk of the sow, thereby increasing their susceptibility to enterotoxigenic Escherichia coli (E. coli) infection and other bacterial, viral and parasitic pathogens^14,15^. We hypothesized that, due to their compromised growth pattern, IUGR piglets would be more susceptible to disease at weaning than normal piglets and this would be measurable in an *ex vivo* blood challenge.

The IUGR piglets weighed 4.5 kg at the end of the experiment (day 24 of age) compared to 6.8 kg for the normal pigs. Over the last decade, weaning age has been dramatically reduced and as a result pigs are smaller and have less mature immune and digestive systems at weaning, making them more susceptible to problems in the early postweaning period^7^. IUGR pigs were approximately one week behind growth wise at day 24 although their fractional growth rate is in fact increased compared to normal pigs^8^. The difference in the immune system between IUGR and normal piglets at day 24 of age was tested using an *ex vivo* LPS challenge model with PBMC to evaluate differences in the ability of the two groups of piglets to respond to an acute inflammatory stimulus. LPS is an integral component of the outer membrane of gram-negative bacteria, which, by stimulating immune cells to synthesise cytokines such as TNFα, IL-1 and IL-6, induce marked changes in behaviour, metabolic and neuroendocrine systems^16^. These cytokine-mediated events are part of a general homeostatic reaction and therefore serve as the first line of defense of the organism against infection^17^.

The majority of both anti- and proinflammatory cytokines were similar for IUGR and normal pigs, with a lower IL-1β in the IUGR group as the only exception. The efficiency of the innate response is crucial for survival and for an optimum priming of acquired immunity^16^. A lower IL-1β response in piglets is probably not beneficial as this would likely mean a slower or diminished response to pathogens such as *E. coli*. The acute phase response is characterized by a dramatic increase in the production of a group of proteins by the liver. Bacterial LPS is an endotoxin, a potent inducer of the acute phase response and systemic inflammation. This response is induced by the production of TNFα, IL-1β, and IL-6 from activated monocytes and neutrophils in response to inflammatory stimuli^18^. This suggests that the immune system of IUGR pigs is not as able as that of normal pigs to mount an innate inflammatory immune response.

For the majority of lymphocyte subsets values were similar for IUGR and normal piglets with only a tendency toward lower percentage for CD3 + CD4+ in IUGR pigs, while IUGR pigs had a significantly lower percentage of total lymphocytes compared to normal piglets. This is most likely due to a drop in CD4+ cells in IUGR pigs compared to normal pigs. A study by Kanitz *et al*.^19^, found that weaning suppresses lymphocyte function, causes changes in neuroendocrine regulation and has a substantial effect on behavioural and endocrine responses to acute peripheral LPS challenge. The results from the current experiment suggest that IUGR piglets are even more susceptible to disease than normal pigs at weaning. Consequently, it could impair health and welfare by increased disease susceptibility in newly weaned piglets^19^. Our hypothesis is therefore partly confirmed, as there was a minor modulation of the immune function in IUGR pigs at day 24 of age, most likely due to a drop in CD4+ t-cells compared to normal piglets.

Total erythrocyte counts were lower in IUGR pigs compared to normal pigs. Erythrocyte levels were found to be lower and levels of pro‐inflammatory cytokines in plasma elevated in humans with periodontitis, potentially suppressing erythropoiesis^20^. Disease is considered to be a major contributing factor associated with poor performance of pigs^7^. Additionally, the weaning transition involves complex social, environmental, and nutritional changes for piglets, and it is a stressful event^6^ and therefore, the results together suggest that IUGR piglets might be more challenged at weaning than normal piglets. Overall, however, the immune phenotype of the IUGR piglets was still broadly similar to the normal piglets, with a few notable differences. A number of other factors are also likely to contribute to the poor health and disease susceptibility of small piglets post-weaning, such as delayed gut development^21^, poor mucosal barrier function^22^, and altered hepatic metabolism^23^.

IUGR pigs had numerically higher levels of reticulocytes and a significant higher level of mean cell volume. Furthermore, the IUGR pigs had fewer erythrocytes but higher levels of MCH compared to normal pigs. Both findings suggest macrocytic anemia in IUGR pigs compared to normal pigs possibly as a compensatory approach in the IUGR pig. In addition, the significant lower levels of IL-1β in IUGR pigs compared to normal pigs suggest that the IUGR pigs are hyporesponsive and cannot produce the same amount of IL-1β as normal pigs. This could suggest that the IUGR pigs are immune compromised and further being hypo responsive is associated with altered bone marrow hematopoiesis. Taken together, the hematological profile of the piglets suggests that IUGR pigs show signs of changes in the bone marrow function compared to normal pigs.

The biochemistry profile was similar between IUGR and normal piglets, suggesting similar protein turnover, enzyme activity and growth in both phenotypes. This further supports growth data from the same farm^10^ where the same growth pattern was found, even though IUGR piglets are 6 days behind compared to normal piglets, mostly due to the smaller starting point. In addition, IGF-1 concentrations were similar between groups^10^. However, Lynegaard *et al*., (2019) did find that the morphometric profile of IUGR piglets at day 24 of age differed from normal piglets. IUGR piglets were found to have relatively larger brain, liver, lungs and adrenal glands at day 24 of age compared to normal piglets^10^. In addition, Huting *et al*.^24^, has also recently shown that morphometric characteristics at birth also affect pig lifetime growth performance. Taken together with the haematological results IUGR piglets are at risk of being delayed both in terms of growth but also due to a slightly suppressed immune response increasing the risk of disease transmission compared to normal piglets.

Only small changes were observed in the TEG analysis. The TEG analysis is a whole-blood, point of care coagulation assay that provides information on the kinetic and mechanical properties of a clot as it forms, matures, and undergoes fibrinolysis^25^. It was hypothesized that TEG results in pigs would identify abnormalities/differences between the groups of pigs and would disrupt hemostasis. In horses TEG performed at admission identified abnormalities associated with inflammatory lesions, systemic inflammatory response syndrome (SIRS), the development of diarrhea, thrombophlebitis, laminitis, and fatality in horses^25^. Although only minor differences were found in the measured TEG parameters, differences were observed in an acute phase protein, fibrinogen, the precursor to fibrin, with higher levels in IUGR pigs.

## Conclusions

In conclusion, a minor modulation of the immune function of IUGR pigs was found at day 24 of age, most likely due to a drop in CD4+ t-cells compared to normal piglets. In addition, a lower level of IL-1β was found suggesting that IUGR pigs are hyporesponsive compared to normal pigs. Furthermore, higher levels of reticulocytes and MCV as well as lower levels of erythrocytes and higher levels of MCH in IUGR pigs suggest altered bone marrow hematopoiesis compared to normal pigs. All together the results suggest that the immune response of IUGR piglets is slightly suppressed compared to normal piglets at day 24 of age. No differences were observed in serum biochemistry supporting a similar growth pattern between IUGR and normal piglets.

## Supplementary information

Supplementary Figure 1 – Gating Strategy for T-cells.

## Data Availability

The datasets used and/or analysed during the current study are available from the corresponding author on reasonable request.
