# Supplementary material for: Intrauterine growth restriction in piglets alters blood cell counts and impairs cytokine responses in peripheral mononuclear cells 24 days post-partum
Source: Sci Rep. 2020 Mar 13;10:4683. doi: 10.1038/s41598-020-61623-w (PMC7069946; doi:10.1038/s41598-020-61623-w)

Intrauterine growth restriction in piglets alters blood cell counts and impairs cytokine responses in peripheral mononuclear cells 24 days post-partum

Charlotte Amdi[1*], Julie C. Lynegaard[1], Thomas Thymann[1] and Andrew R. Williams[1]

[1]Department of Veterinary and Animal Sciences, Faculty of Health and Medical Sciences, University of Copenhagen, DK-1870 Frederiksberg C, Denmark

[*]Corresponding author: Charlotte Amdi Williams; e-mail: ca@sund.ku.dk

Short title: LPS challenge in IUGR and normal pigs

Supplementary Figure 1 – Gating Strategy for T-cells

PBMCs were gated on forward/side scatter and CD3+ cells gated. CD4+ CD8+ single and double positive cells were then identified.

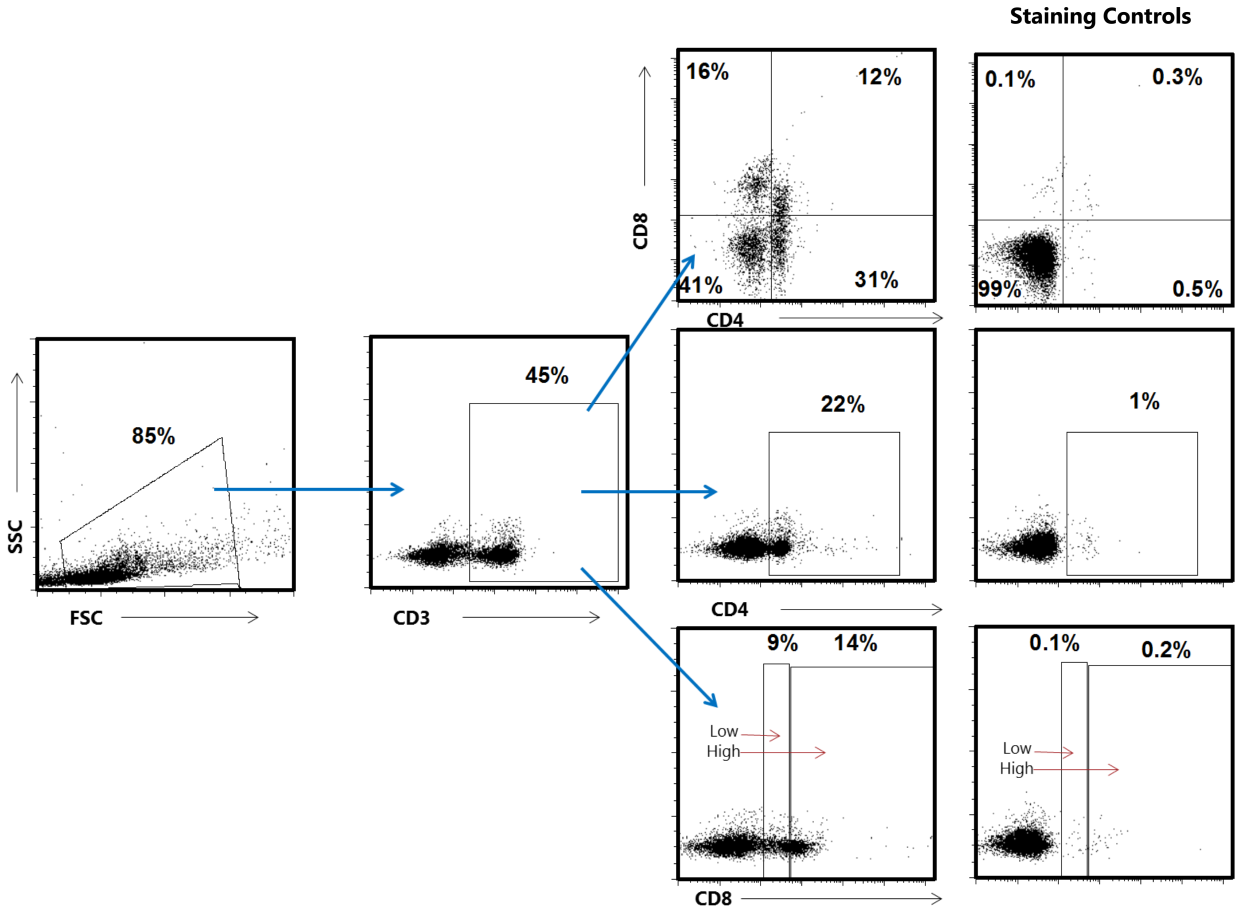

Supplement: Supplementary file 1 — Supplementary Figure 1 – Gating Strategy for T-cells. [file 41598_2020_61623_MOESM1_ESM.pdf]
